# Impairment of DHA synthesis alters the expression of neuronal plasticity markers and the brain inflammatory status in mice

**DOI:** 10.1096/fj.201901890RR

**Published:** 2019-12-11

**Authors:** Emanuela Talamonti, Valeria Sasso, Hoi To, Richard P. Haslam, Johnathan A. Napier, Brun Ulfhake, Karin Pernold, Abolfazl Asadi, Tara Hessa, Anders Jacobsson, Valerio Chiurchiù, Maria Teresa Viscomi

**Affiliations:** ^1^ Department of Biochemistry and Biophysics Stockholm University Stockholm Sweden; ^2^ Department of Molecular Biosciences The Wenner‐Gren Institute Stockholm University Stockholm Sweden; ^3^ Laboratory of Experimental Neurorehabilitation IRCCS Santa Lucia Foundation Rome Italy; ^4^ Department of Plant Science Rothamsted Research Harpenden UK; ^5^ Department of Neuroscience Karolinska Institute Stockholm Sweden; ^6^ Department of Medicine Campus Bio‐Medico University of Rome Rome Italy; ^7^ Laboratory of Resolution of Neuroinflammation IRCCS Santa Lucia Foundation Rome Italy; ^8^ Istituto di Istologia ed Embriologia Università Cattolica del S. Cuore Rome Italy

**Keywords:** anti‐inflammatory molecules, brain plasticity, microglia, omega‐3, PUFA

## Abstract

Docosahexaenoic acid (DHA) is a ω‐3 fatty acid typically obtained from the diet or endogenously synthesized through the action of elongases (ELOVLs) and desaturases. DHA is a key central nervous system constituent and the precursor of several molecules that regulate the resolution of inflammation. In the present study, we questioned whether the impaired synthesis of DHA affected neural plasticity and inflammatory status in the adult brain. To address this question, we investigated neural and inflammatory markers from mice deficient for ELOVL2 (Elovl2^−/−^), the key enzyme in DHA synthesis. From our findings, Elovl2^−/−^ mice showed an altered expression of markers involved in synaptic plasticity, learning, and memory formation such as Egr‐1, Arc1, and BDNF specifically in the cerebral cortex, impacting behavioral functions only marginally. In parallel, we also found that DHA‐deficient mice were characterized by an increased expression of pro‐inflammatory molecules, namely TNF, IL‐1β, iNOS, caspase‐1 as well as the activation and morphologic changes of microglia in the absence of any brain injury or disease. Reintroducing DHA in the diet of Elovl2^−/−^ mice reversed such alterations in brain plasticity and inflammation. Hence, impairment of systemic DHA synthesis can modify the brain inflammatory and neural plasticity status, supporting the view that DHA is an essential fatty acid with an important role in keeping inflammation within its physiologic boundary and in shaping neuronal functions in the central nervous system.

**Abbreviations**
Arc1: activity‐regulated cytoskeleton‐associated protein 1
BDNF: brain‐derived neurotrophic factor
DHA: docosahexaenoic acid
Egr‐1: early growth response protein 1
ELOVLs: elongases
Elovl2: elongation of long‐chain fatty acids 2
EPA: alpha‐linolenic acid‐derived eicosapentaenoic acid
GFAP: glial fibrillary acidic protein
Iba1: ionized calcium‐binding adapter molecule 1
PUFA: ω‐3 polyunsaturated fatty acids
SPMs: specialized pro‐resolving mediators
TMEM‐119: transmembrane protein 119

## 1 INTRODUCTION

The main ω‐3 polyunsaturated fatty acid (PUFA) of the brain is docosahexaenoic acid (DHA), which is a key structural component of neuronal membrane phospholipids and plays an essential role in brain development and function.^1^, ^2^ DHA cannot be synthesized de novo in mammals and need to be synthesized from the essential dietary fatty acid α‐linolenic acid (ALA) through a series of elongation and desaturation reactions performed by distinct enzymes residing in the endoplasmic reticulum.^3^ Indeed, DHA biosynthesis requires three elongation steps, the last two of which are catalyzed by elongation of long‐chain fatty acids 2 (ELOVL2), an enzyme that is significantly expressed in liver, testis, retina and central nervous system (CNS), tissues that are particularly rich in DHA.^4^, ^5^, ^6^

DHA is essential for CNS functions, including neurite outgrowth and synaptogenesis,^7^ brain‐derived neurotrophic factor (BDNF)‐induced neuronal survival,^8^ synaptic integrity and neurotransmission^9^ as well as protection from neuroinflammatory processes, and cognitive decline.^10^ Indeed, a DHA deficient diet is involved in the pathophysiology of memory impairment and several mood disorders such as anxiety and depressive‐like behaviors.^11^, ^12^, ^13^, ^14^, ^15^ DHA deficiency in the CNS is also paralleled by an increased expression of pro‐inflammatory cytokines and a modification of neuronal plasticity‐related gene expression, including the early growth response protein 1 (Egr‐1) and the activity‐regulated cytoskeleton‐associated protein (Arc‐1),^16^ ultimately associated with learning and memory formation.^17^, ^18^ Such evidence prompted the investigation of several clinical trials with DHA on neurodevelopmental disorders, including autism and attention‐deficit/hyperactivity disorder.^19^ Despite the fact that there is a growing number of trials testing the efficacy of n‐3 PUFAs in the treatment of different psychiatric disorders, only a few studies investigated the efficacy of DHA alone on cognitive functions. Moreover, the small number of trials on cognitive impairment in psychiatric disorders shows doubtful and/or contrasting results that need further investigations.^20^, ^21^

Moreover, the role of DHA in inflammation is already consolidated and is receiving greater attention mainly due to the discovery that it serves as a metabolic precursor for a new genus of anti‐inflammatory molecules, that is, resolvins, neuroprotectins, and maresins, that are involved in the spontaneous but active process of resolution of inflammation,^21^, ^22^ which mainly involves myeloid cells such as macrophages and glial cells.^23^, ^24^ Indeed, we have recently shown that the impairment of systemic DHA synthesis in Elovl2^−/−^ mice, delineates an immunophenotypical alteration of M1/M2 macrophages, with M1 being more pro‐inflammatory and M2 less protective, supporting the view that DHA has a relevant role in controlling inflammatory responses.^25^ Given that DHA plays a key role in CNS function and plasticity and is a potent anti‐inflammatory mediator, we sought to systematically investigate whether Elovl2^−/−^ mice, characterized by a systemic impairment of endogenous DHA synthesis, displayed neuronal plasticity dysfunctions and brain inflammation and whether such alterations could be restored upon DHA supplementation.

## 2 MATERIAL AND METHODS

### 2.1 Ethics statement for experimental animals

This study was approved by the Institutional Review Board on Animal Studies of Stockholm University and by the Animal Ethics Board of the North Stockholm region, and performed in accordance with national guidelines and regulations for the care and use of laboratory animals in agreement with the guidelines of the European Communities Council Directive 2010/63/EU for the care and use of laboratory animals. All efforts were made to minimize the number of animals used and their suffering.

### 2.2 Animals and tissue processing

Elovl2^−/−^ mice were generated as described previously^26^ and backcrossed into the 129S2/Sv strain for five generations. All animals were housed at room temperature and maintained on a 12 hours light/dark cycle. About 20‐25 weeks old male or female mice were fed standard chow DHA‐free diet (10% kcal fat, D12450H, Research Diets, New Brunswick, NJ, USA) or DHA‐enriched (10% kcal fat, 1% DHA, D13021002, Research Diets, New Brunswick, NJ, USA) for 3 months, according to the experimental groups. Dietary fatty acid composition and other nutrients are shown in Tables 1 and 2. All animals were fed ad libitum and had free access to water. At the end of the study, animals were euthanized with CO_2_ and killed by cervical dislocation. Brains were removed immediately, dissected, and stored in liquid nitrogen or in 4% paraformaldehyde.

**Table 1:** Diet fatty acid composition

Fatty acids	not DHA‐enriched diet (10% kcal fat) D12450H	DHA‐enriched diet (10% kcal, 1% DHA) D13021002
C12:0	0.09	0.05
C14:0	0.80	0.48
C15:0	0.05	0.05
C16:0	16.83	12.48
C16:1n‐9	0.17	0.09
C16:1n‐7	0.86	0.51
C18:0	8.43	5.35
C18:1n‐9	27.87	21.35
C18:1n‐7	1.66	1.29
**C18:2n‐6** a	**37.43**	**35.64**
C18:3n‐6	0.16	0.19
**C18:3n‐3** b	**4.24**	**4.46**
C20:0	0.30	0.23
C18:4n‐3	0.05	0
C20:1n‐9	0.37	0.25
C20:2n‐6	0.31	0.19
C20:4n‐6	0.11	0.24
C22:0	0.24	0.20
C20:4n‐3	0	0.03
C20:5n‐3	0	0.71
C22:3n‐3	0	0.08
C22:4n‐6	0	1.04
C22:5n‐3	0	0.57
**C22:6n‐3** c	**0**	**14.53**

Note

Fatty acids expressed as % of total fatty acids of control diet (10% kcal fat, D12450H, Research Diets, New Brunswick, NJ, USA) and DHA‐enriched diet (10% kcal fat, 1% DHA, D13023002, Research Diets, New Brunswick, NJ, USA).

a LA (linoleic acid).

b ALA (α‐linolenic acid).

c DHA (docosahexaenoic acid).

**Table 2:** Diet composition

Ingredient	Control Diet (10% kcal fat) D12450H	DHA‐Enriched Diet (10% kcal fat, 1% DHA) D13021002
Casein	200	200
L‐cystine	3	3
Cornstarch	452.2	452.2
Maltodextrin 10	75	75
Sucrose	172.8	172.8
Cellulose, BW200	50	50
Soybean oil	25	25
**DHA**	**0**	**10**
Lard	20	10
Mineral mix S10026	10	10
Dicalcium phosphate	13	13
Calcium carbonate	5.5	5.5
Potassium citrate, 1H20	16.5	16.5
Vitamin mix V10001	10	10
Choline bitartrate	2	2
FD&C yellow dye #5	0.04	0
FD&C red dye #40	0.01	0.025
FD&C blue dye #1	0	0.025
Total (g)	1055.5	1055.05

Note

Diet composition expressed in gram of mass for each ingredient, of control diet (10% kcal fat, D12450H, Research Diets, New Brunswick, NJ, USA) and DHA‐enriched diet (10% kcal fat, 1% DHA, D13023002, Research Diets, New Brunswick, NJ, USA) formulated by Research Diets, New Brunswick, NJ, USA.

### 2.3 HPLC analysis of acyl‐CoA fatty acids

Brain tissues were ground to a powder and 6 mg extracted^27^ for reverse‐phase LC (Agilent 1200 LC system; Gemini C18 column, 2 mm inner diameter, 150 mm with 5 mm particles) with electrospray ionisation tandem mass spectrometry (multi reaction monitoring, MRM) on a QTRAP 4000 (SCIEX) instrument in positive ion mode. LC‐MS/MS MRM analysis followed the methods previously described.^28^ A total of 68 acyl‐CoA fatty acids (14:0 to 34:6) were tracked using individual MRM mass pairs. For identification and calibration, standard acyl‐CoA esters with acyl chain lengths from C14 to C20 were purchased from Sigma as free acids or lithium salts.

### 2.4 Western blotting

Whole brains and the cerebral cortex were collected, homogenized, and proteins were extracted in Ripa buffer (Milli‐Q Water, Tris hydrochloride 0.05 M pH 7.4, 0.001 M potassium chloride, 0.0015 M magnesium chloride, 0.001 M EDTA, 0.001 M dithiothreitol, 0.005 M sodium fluoride, 0.001 M sodium metavanadate, 0.1% SDS, 10% Na‐DOC,1% Triton X‐100, 1X protease inhibitor cocktail (Sigma, P8340), for 20 minutes on ice, and then were centrifuged for 15 minutes at 4°C (14 000 rpm). Supernatants were collected and transferred in a 1.5 mL vial. The total protein content of the resulting supernatant was quantified by the Bradford's colorimetric assay (Bio‐Rad, Milan, Italy). Each protein sample was separated by SDS‐polyacrylamide gel electrophoresis and transferred to a nitrocellulose membrane. Membranes were saturated with 5% dried nonfat milk and incubated overnight at 4 degrees C with specific primary antibodies. Membranes were then incubated with the appropriate horseradish peroxidase‐conjugated secondary antibodies. Immunoreactive bands were detected using an enhanced chemiluminescence kit (ECL; Amersham Biosciences). The relative levels of immunoreactivity were determined by densitometry using the software ImageQuant 5.0. Samples were incubated with the following primary antibodies: rabbit anti‐Caspase 1 (1:1000, Cell Signaling, Danvers, MA, USA); monoclonal mouse anti‐Arc (1:200, Santa Cruz Biotechnology, Dallas, TX, USA), rabbit anti‐Iba1 (1:500, Wako, Japan), rabbit anti‐GFAP (1:1000, #AB5804 Merck Millipore, Burlington, MA, USA), mouse anti‐actin (1:1000, Cell Signaling), mouse anti‐Egr‐1 (1:500, Santa Cruz Biotechnology), and mouse anti‐BDNF (1:500, Immunological Science, Rome, Italy). Densities of protein bands in the Western blots were measured with ImageJ and mean ratios between proteins and actin were reported as a percentage of control values.

### 2.5 qRT‐PCR

Total RNA was isolated with TRI Reagent (Sigma‐Aldrich) and total RNA was isolated following the manufacturer's procedure. For real‐time PCR, 500 ng total RNA was reverse transcribed using random hexamer primers, deoxynucleoside triphosphates, MultiScribe reverse transcriptase, and RNase inhibitor (Applied Biosystems, Foster City, CA). cDNA samples were diluted 1:10 and aliquots of 2 μl per reaction were run in duplicate. Thermal cycling conditions were 2 minutes at 50°C, 10 minutes at 95°C, and 40 cycles of 15 s at 95°C and 1 minutes at 60°C, followed by the melting curve analysis on a Bio‐Rad CFX Connect Real‐Time system. Primers used for qRT‐PCR were caspase‐1, inducible nitric oxide synthase (iNOS), interleukin 1β (IL‐1β), tumor necrosis factor (TNF), activity‐regulated cytoskeleton‐associated protein 1 (Arc‐1), early growth response protein 1 (Egr‐1), brain‐derived neurotrophic factor (BDNF), and glial fibrillary acidic protein (GFAP). Actin and transcription factor IIB (TFIIB) were used as housekeeping genes for quantity normalization. For primers’ sequences see Table 3.

**Table 3:** Murine primers used for the quantitative RT‐PCR

*Primers*	Forward	Reverse
*Casp1*	GGACATCCTTCATCCTCAGAAACA	TTTCTTTCCATAACTTCTGGGCTTT
*iNOS*	TCTCCCTTTCCTCCCTTCTT	CTTCAGTCAGGAGGTTGAGTTT
*IL‐1β*	TGCCACCTTTTGACAGTGATG	AAGGTCCACGGGAAAGACAC
*TNF*	CCACATCTCCCTCCAGAAA	CTTCTGCCAGTTCCACGTC
*Egr‐1*	AGCGAACAACCCTATGAGCA	ATAACTCGTCTCCACCATCCGC
*BDNF*	TGGCATCTACCCACACACTTT	TCAGTTTGTTCGGCTCCACT
*GFAP*	GGCCCTGAGAGAGATTCGC	ACCGATACCACTCCTCTGTGCT
*Arc‐1*	ACGATCTGGCTTGGTCATTCTGG	AGGTTCCCTGAGCATGTCTGCTT
*TFIIB*	TGGAGATTTGTCCACCATGA	GAATTGCCAAACTCATCAAAACT
*B‐actin*	AGTCCCTGCCCTTTGTACACA	CGATCCGAGGGCCTCACTA

### 2.6 Behavioral testing

Behavioral testing took place during a fixed time window from 1 PM to 5 PM, depending on a number of tests. This is toward the end of the light cycle (6 AM to 6 PM). About 28 mice at 6‐10 months of age were used for behavioral testing. Males and females were always tested separately and all maintained on a standard diet. In all nonautomated test recordings, two observers were present. Observers were placed in a fixed position in each test, for example, on opposing sides of a mesh grid. Prior to testing, all animals had at least one week of acclimatization following rehousing or arrival to the animal house. For the open field activity, ambulation levels as well as anxiety were examined with the open field test described in,^29^ using a square area with light grey floor and clear Plexiglass walls (50 × 50 × 38 cm surrounded by to frames of light beams; at 3 cm above the arena floor to detect movements and at 6 cm above the arena floor to detect rearing events (ActiMot system, TSE systems, Germany). The light level in the center of the arena floor was adjusted to 150‐200 lux. Explorative behavior was recorded for 90 minutes. During this period the following behavioral characteristics were evaluated: a) ambulation; b) rearing; c) immobility. For the novel object recognition (NOR) experiments, mice (n = 7 per experimental group) were acclimatized in a 29 × 29 cm square cage (arena) made of yellow “plastic” with 35 cm high walls for 5 minutes just prior the training. During the training trial, two identical plastic objects with particular shapes and colors were presented. The mouse was allowed to explore both objects for a cumulative time of 10 seconds (cut‐off time 15 min). After a 24 hours inter‐trial interval (ITI), one of the familiar objects was replaced by a novel object, with a different shape and color, to test for memory retention. The mouse was again allowed to explore both objects for a cumulative time of 10 seconds (cut‐off time 15 min) and exploration of an object was defined as pointing the nose to the object (within 1 cm distance from the object) and/or touching the object with the nose. Analysis was based on the time spent exploring the novel and the familiar objects.^29^ For the Barnes maze test, we tested only female animals. This test occurs on a circular platform (Ø125 cm, elevated 70 cm above the floor) with 36 escape holes ringed around the center of the platform. Bright overhead lighting creates an aversive stimulus, encouraging the animal to escape the light through the Target Escape Hole, which is attached to an escape box. Visual cues placed around the maze act as spatial cues. In this test, we used a shortened protocol developed by Attar et al,^30^ with five training trials over 2 days followed by the probe trial on the third day. This protocol emphasizes the early learning phase and is more sensitive with respect to discrete spatial learning difficulties/memory consolidation than the standard protocol with 15 training trials. The only aversive stimulus used was light (approx. 400‐500 lux in the center of the arena). All tests were recorded with a high‐resolution video camera at 15 frames per second and analyzed with the Ethovision XT 11 software (Noldus IT, The Netherlands).

### 2.7 Histology and immunohistochemistry

Mice were perfused transcardially with 20 mL of saline followed by 20 mL of 4% paraformaldehyde in phosphate buffer (PB; 0.1 M; pH 7.4) under anesthesia induced by  i.p.  injection of sodium pentobarbital (60 mg/kg). Each brain was removed immediately, postfixed in the same fixative for 2 hours and, after three washes in PB, transferred to 30% sucrose in PB solution at 4°C until they sank. Brains were cut into four series of 30 μm‐thick transverse sections by means of a freezing microtome and were collected in PB. The sections were incubated overnight with a cocktail of primary antibodies, one of this including rabbit anti‐GFAP (1:1000; #Z0334, Dako Agilent, Santa Clara, CA, USA), rabbit anti‐Iba1 (1:700; Wako, Japan), goat anti‐Iba1 (1:400; Abcam, Cambridge, MA, USA), rabbit anti‐TMEM‐119 (1:400; Abcam), goat anti‐IL‐1β (1:200; R&D Systems, Minneapolis, MN, USA), and goat anti‐TNF (1:200; R&D Systems, Minneapolis, MN, USA). All primary antibody solutions were prepared in PB and 0.3% Triton X‐100 and were incubated overnight. Afterward, sections were incubated for 2 hours at RT with a cocktail of secondary antibodies, including Alexa Fluor 488 conjugated donkey anti‐mouse (1:200; Thermo Fisher Scientific, Waltham, MA USA), Alexa Fluor 555 conjugated donkey anti‐rabbit (1:200), Alexa Fluor 488 conjugated donkey anti‐rabbit (1:200), and Alexa Fluor 555 conjugated donkey anti‐goat (1:200). Sections were also counterstained with Neuro‐Trace 640⁄660 deep‐red Fluorescent Nissl Stain (1:200; Thermo Fisher Scientific). After further washes in PBS, the sections were mounted using an anti‐fade medium (Fluoromount; Sigma, Milan, Italy) and examined under a confocal laser‐scanning microscope (Zeiss CLSM700, Oberkochen Germany). The specificity of immunohistochemical labeling was confirmed by the omission of primary antibodies and the use of normal serum instead (negative controls). Confocal acquisition of the brain area of interest (motor cortex and somatosensory cortex) was identified by a 10 × objective (Plan‐Apochromat, Zeiss; NA = 0.30; zoom factor 0.5) and captured through a confocal laser‐scanning microscope (CLSM700; Zeiss, Oberkochen, Germany). The following acquisition settings were used: image format 1024 × 1024; image size 1279.1 × 1279.1 μm; Airy Units 1.0 producing an optical section thickness of 5.2 μm; pixel dwell time 12.61 μs. The confocal image acquisitions were performed so that all samples were captured using consistent settings for laser power and detector gain. Images were exported in the TIFF format, contrast and brightness were adjusted only during image processing for counting to ensure that fluorescence intensity of Iba1 staining falls within the linear range for each sample to provide uniform visualization of cells. Final plates were composed with Adobe Illustrator CS3.

### 2.8 Quantitative analysis of microglia

To assess microglia and astrocytes proliferation in the cerebral cortex (motor cortex and sensorimotor cortex), quantitative analyses were performed off‐line on confocal images acquired through the 10x objective at the 0.7 zoom factor. Specifically, all labeled cells in two rectangular boxes (100 µm width × 200 µm length) randomly positioned in five regularly spaced sections were counted. Iba1 or GFAP‐immunolabeled cells were digitally marked and recorded, and data were stored in an archive as previously described.^31^

To assess microglial morphological features, quantitative analyses were performed on‐line on sections stained with Iba1 and counterstained with Neuro‐Trace to identify the areas of interest. Sections were visualized using an optical microscope (DMLB, Leica, Wetzlar, Germany) equipped with a motorized stage and a camera connected to software (Neurolucida 7.5, MicroBrightField). Iba1 positive cells were reconstructed under a Zeiss Axio Plan objective (NEOFLUAR × 100 objective with a numerical aperture of 1.3 under oil immersion). The only microglia that displayed intact processes unobscured by background labeling or other cells were included in reconstructions. Microglia were traced throughout the entire thickness of the section (30 μm) to acquire morphometric analysis of the cell body (cross‐sectional area and perimeter) and the processes ramification (number of branch nodes and intersections) (Table 4). To account for changes in the cell's complexity in relation to distance from the cell soma, Sholl analysis was performed for each cell. Concentric circles (radii) were spaced 10 μm apart, originating from the soma. The number of branch points (nodes), processes that intersected the radii, was measured as a function of the distance from the cell soma for each radius.^24^, ^32^ Data were collected and stored for statistical analysis. Fifteen cells per animal were randomly selected for a total of 90 cells per group (N = 6 mice/group) and included for analysis. All quantitative analyses were conducted blind to the animal's experimental group assignment.

**Table 4:** Summary of microglia morphology measures

	Unit of measure	Sampling	Interpretation
Perimeter of the cell body	µm	15 cells per animal	Cell body size
Cross‐sectional area of the cell body	µm^2^	15 cells per animal	Cell body size
Processes intersection (with each radius)	Number (per radius)	15 cells per animal	Cell ramification
Branch points (nodes) for each radius	Number (per radius)	15 cells per animal	Cell ramification

### 2.9 ELISA assay

The cytokine content was determined by standard two‐site sandwich ELISAs, using the available commercial kits for IL‐1β (detection limit 2 pg/mL) (eBioscience, ready‐set‐go), according to manufacturer's instructions.

### 2.10 Statistical analysis

Data were expressed as mean ± SEM and were analyzed by means of Prism 4 software (GraphPad Software, San Diego, CA). Differences between two or multiple groups were analyzed by means of the Student's t test or one‐way ANOVA followed by Bonferroni *post hoc* test or Tukey's test. A *P* value <.05 was considered significant. Since there were no gender‐related differences, data from male and female mice were pooled together. For each experiment, except for behavioral studies, 40% males and 60% females were randomized between the groups.

## 3 RESULTS

### 3.1 DHA deficiency affects fatty acid composition in adult brains

**Figure 1:**
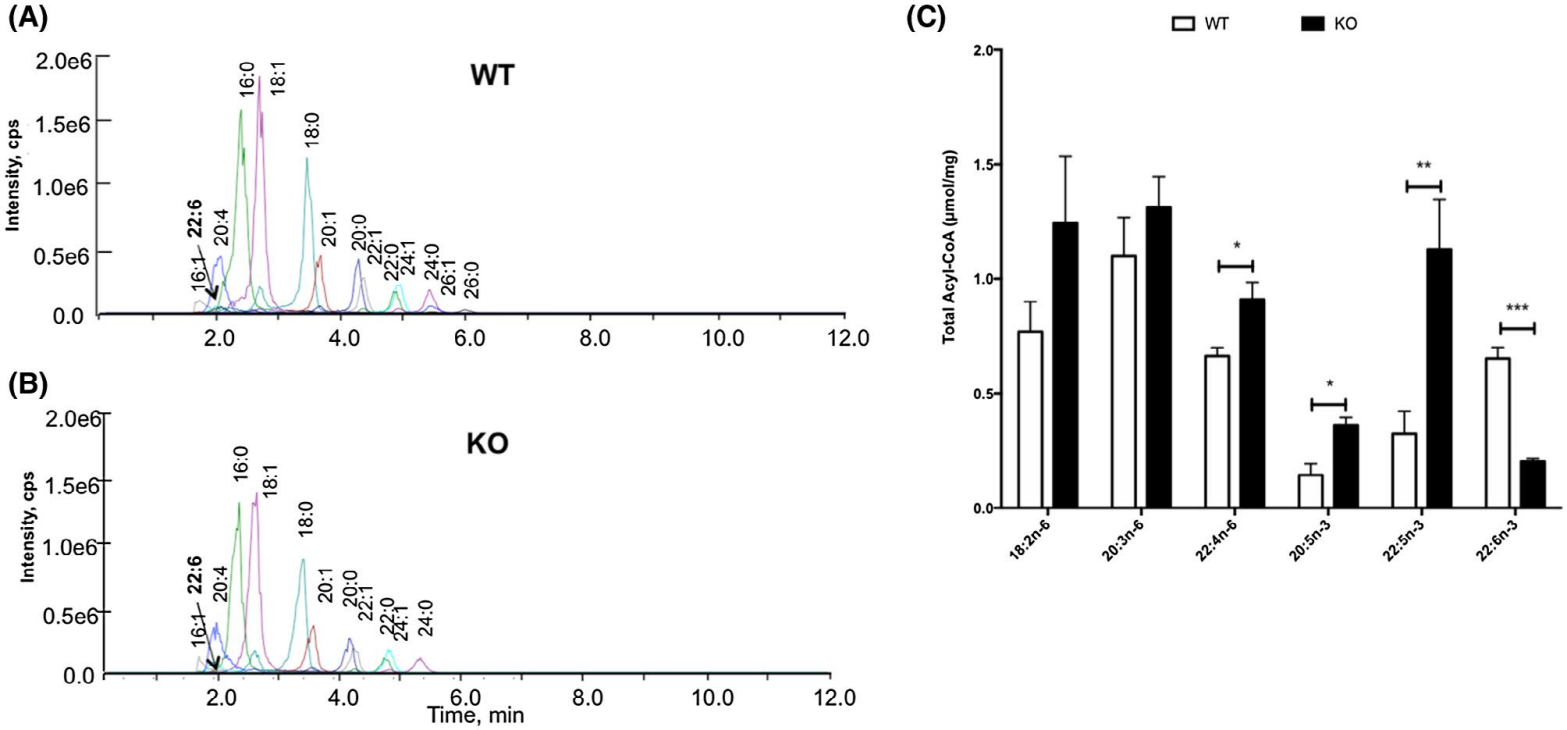
Biosynthesis of fatty acids in wild‐type (WT) and Elovl2^−/−^ (KO) mice brains. A‐B, Representative spectra of WT and KO mice fed standard chow diet (no DHA) and determined by extraction, derivatization, and LC‐MS/MS analysis of acyl‐etheno‐CoAs fatty acids. C, Composition of key n‐6 and n‐3 fatty acids in the brain of WT and KO. Results shown are means ± SEM from seven animals per group. *P < .05, **P < .01, and ***P < .001

To directly link PUFA synthesis in the brain with alterations in brain plasticity and inflammation, we profiled for the first time the acyl‐CoA composition of various fatty acids in the brain tissues of Elovl2^−/−^ (KO) and wild‐type (WT) mice using targeted metabolite monitoring via liquid chromatography in combination with mass spectrometry (LC‐MS/MS) (Figure 1A). Such lipidomic analysis allowed us to detect several fatty acids derived both from alpha‐linolenic acid (n‐3) and linoleic acid (n‐6), namely the metabolic pool in which the Elovl enzymes work. In particular, although most fatty acids were detectable in both WT and KO mice (Figure 1A,B), we observed a significant accumulation of alpha‐linolenic acid‐derived eicosapentaenoic acid (EPA) (20:5n‐3) and docosapentaenoic acid (DPA) (22:5n‐3) coupled with, as expected, markedly reduced the levels of DHA (22:6n‐3) in the brain of KO mice (Figure 1C). Interestingly, since Elovl2 is also involved in the elongation of linoleic acid‐derived n‐6 fatty acids, KO mice displayed a general, yet not significant, accumulation of linoleic acid itself (18:2n‐6) and dihomo‐γ‐linoleic acid (20:3n‐6), and a significant accumulation of docosatetraenoic acid (20:5n‐6) (Figure 1C). Furthermore, when looking at other n‐3 and n‐6 fatty acids, such as 20:3n‐3, 20:4n‐3 or 22:4n‐6, we did not observe any significant change between WT and KO brain tissues (Table 5). Similarly, also several saturated fatty acids, such as arachidic acid (20:0), behenic acid (22:0), and lignoceric acid (24:0), were unchanged between WT and KO brains, with the exception of stearic acid (18:30) that was significantly reduced in KO brains (Table 5).

**Table 5:** Composition of other n‐6, n‐3, and saturated fatty acids in the brain of WT and KO

Acyl‐CoA fatty acid	WT (μmol/mg)	KO (μmol/mg)
C12:0	15.98 ± 0.89	14.21 ± 0.59
C14:0	5.73 ± 0.55	6.06 ± 0.68
C15:0	1.01 ± 0.17	1.13 ± 0.22
C16:0	9.55 ± 1.65	11.07 ± 1.23
C16:1n‐9	3.74 ± 0.80	3.86 ± 0.75
C16:1n‐7	0.66 ± 0.04	0.85 ± 0.08
C18:0	4.45 ± 0.98	4.72 ± 0.95

Note

Results shown are means ± SEM from seven animals per group.

* *P* < .05.

### 3.2 DHA deficiency affects synaptic plasticity genes

**Figure 2:**
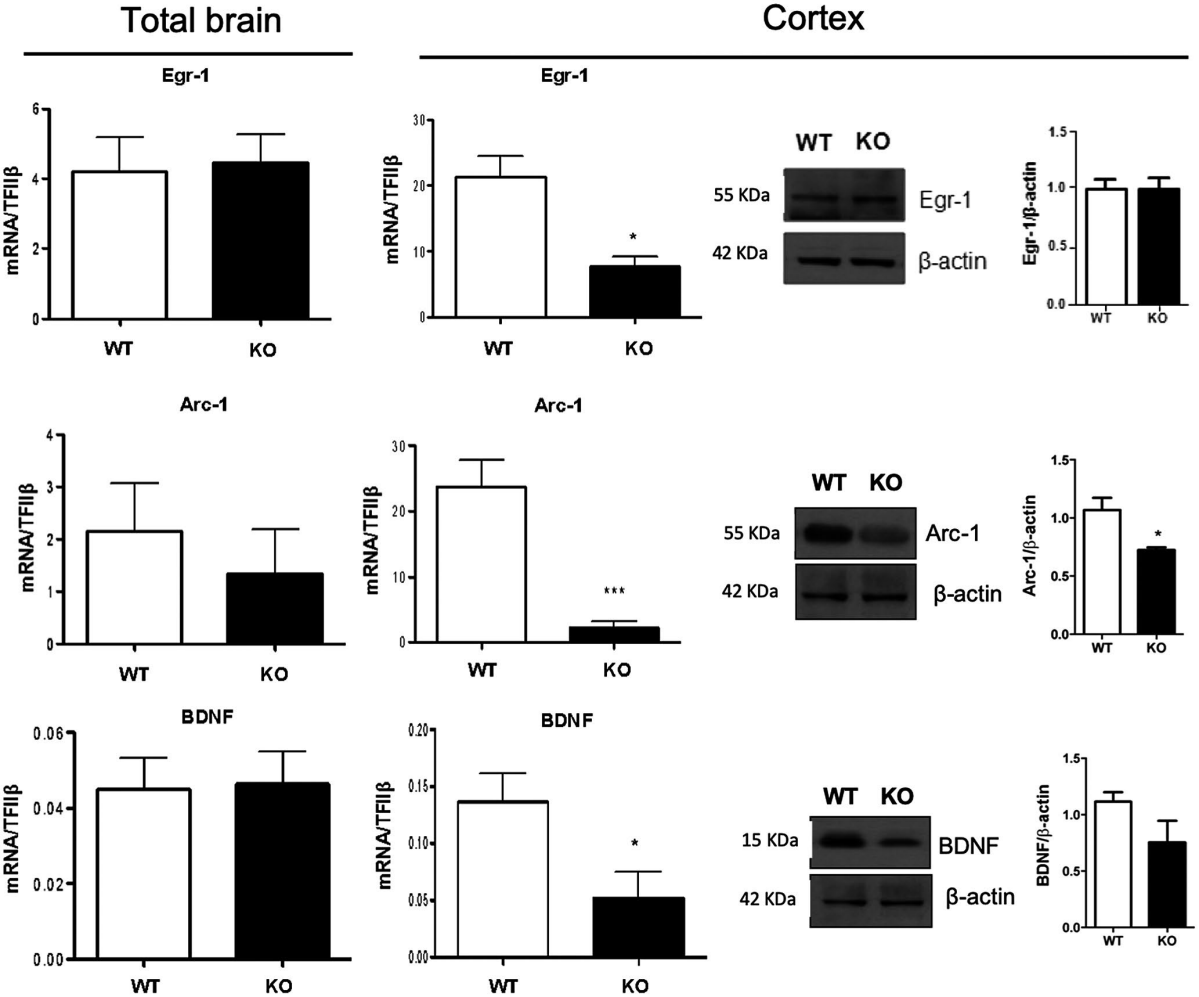
Synaptic plasticity markers expression is impaired in the cerebral cortex of KO mice. mRNA expression of Egr‐1, Arc‐1, and BDNF in the total brain and their mRNA and protein in the cortex of WT and KO mice by RT‐PCR and western blotting. Data are shown as mean ± SEM of seven animals per group (WT and KO), each in duplicate. *P < .05 and ***P < .001 vs WT by unpaired Student's t test

Next, we questioned whether several markers of brain plasticity were affected following impaired DHA synthesis. To do so, we analyzed the expression of several well‐known plasticity factors that are rapidly and selectively regulated in specific brain regions associated with learning and memory, including Arc‐1, Egr‐1, and BDNF.^33^, ^34^ Considering that the expression of these factors has previously been shown to be regulated by ω‐3 polyunsaturated fatty^35^, ^36^ we measured the gene expression of Arc‐1, Egr‐1, and BDNF in the total brain and cerebral cortex. Interestingly, the expression of these neuroplasticity immediate early genes did not show any significant variation in the total brains of KO mice compared to WT (Figure 2). However, when analyzing them in the cerebral cortex, we observed that the mRNA expression of Egr1 and its direct target Arc1, as well as of BDNF was significantly down‐regulated in KO mice compared to WT (Figure 2). When trying to corroborate such findings in the cortex by analyzing their protein levels, we observed that only Arc‐1 was significantly reduced in KO mice compared to WT, whereas no significant change was observed for BDNF and Egr‐1 (Figure 2).

### 3.3 DHA deficiency induces neuroinflammation but has a small impact on behavioral functions

**Figure 3:**
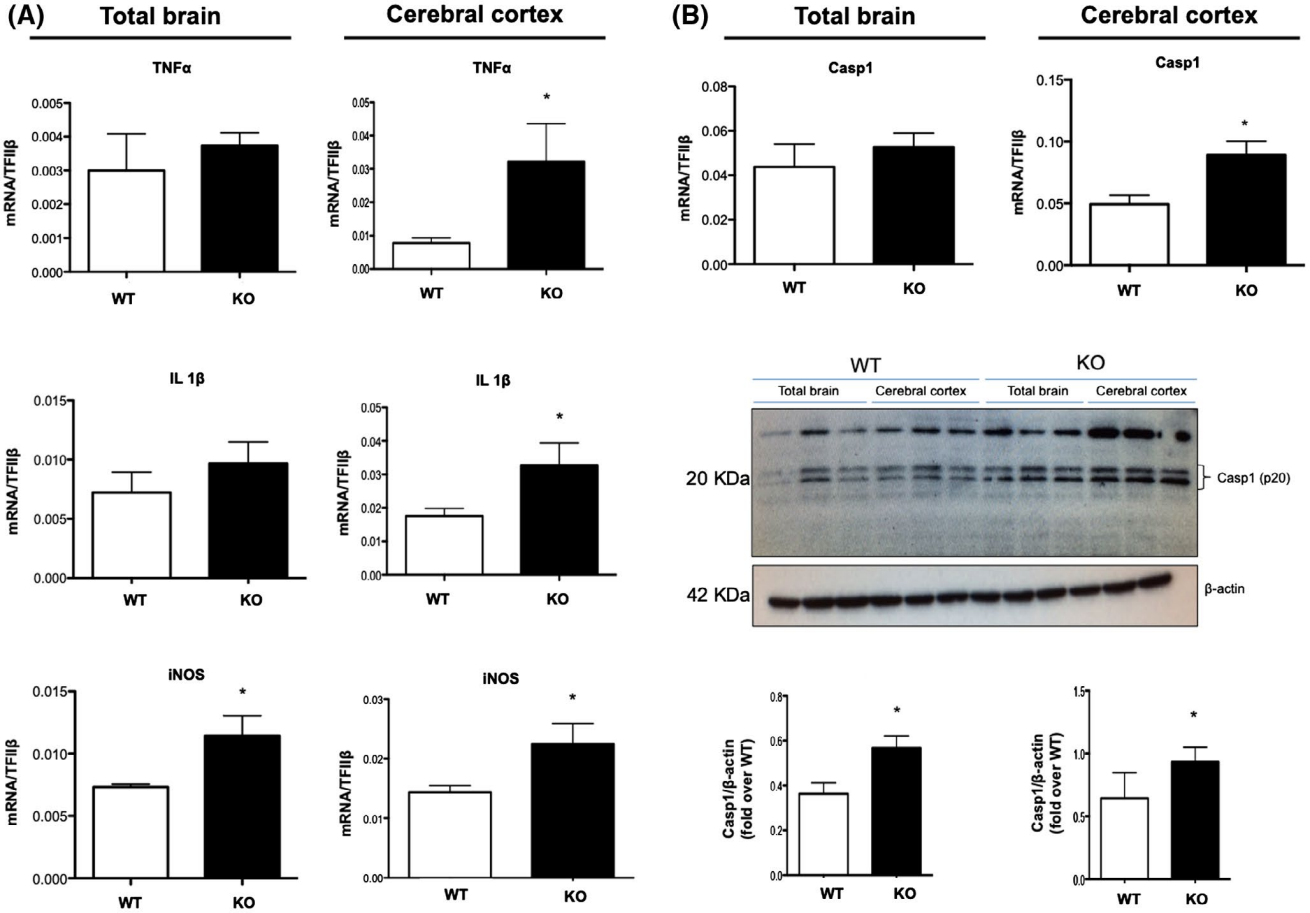
Inflammatory markers expression is impaired in the cerebral cortex of KO mice. A‐B, mRNA expression of TNF, IL‐1β, iNOS, and Casp1 as well as protein expression of Casp1(p20) in total brain and cortex of WT and KO mice by qRT‐PCR and western blotting. Data are shown as mean ± SEM of seven (qRT‐PCR) or four (western blotting) animals per group (WT and KO), each in duplicate. *P < .05 vs WT by unpaired Student's t test

As already shown by our previous studies where impairment of systemic DHA synthesis affects specific immune cells, such as T‐helper cells and macrophages^37^, ^38^ and given the important role of DHA in inflammatory responses, we next examined whether the deficient synthesis of this essential fatty acid can modulate the brain inflammatory status. For this purpose, we examined the expression of several markers of brain inflammation in total brain and cerebral cortex of KO and WT mice, among which the major pro‐inflammatory cytokines in the brain (TNF and IL‐1β), inducible nitric oxide synthase (iNOS), and caspase‐1 (Casp1), all of which play a key role in the onset and progression of neuroinflammatory diseases.^39^ In particular, the total brains of KO mice showed a significant increase in iNOS gene expression but no variation in TNF, IL‐1β, and Casp1 (Figure 3A,B) compared to WT mice. However, Casp1 protein expression was significantly higher in KO mice compared to WT (Figure 3B). Conversely, when investigating the expression of these pro‐inflammatory markers in the cerebral cortex, we observed that mRNAs of both cytokines, as well as iNOS and Casp1, were significantly up‐regulated in KO mice. Since Casp1 is usually in its physiologically inactive zymogen form and requires cleaving into its p20 subunit to display its pro‐inflammatory activity,^40^ we also analyzed the protein expression of its biologically active fragment p20 in WT and KO mice and we found that KO mice showed a significant increase in p20‐casp1 expression compared to WT mice (Figure 3A,B), thus confirming a higher inflammatory status in mice deficient for DHA synthesis.

**Figure 4:**
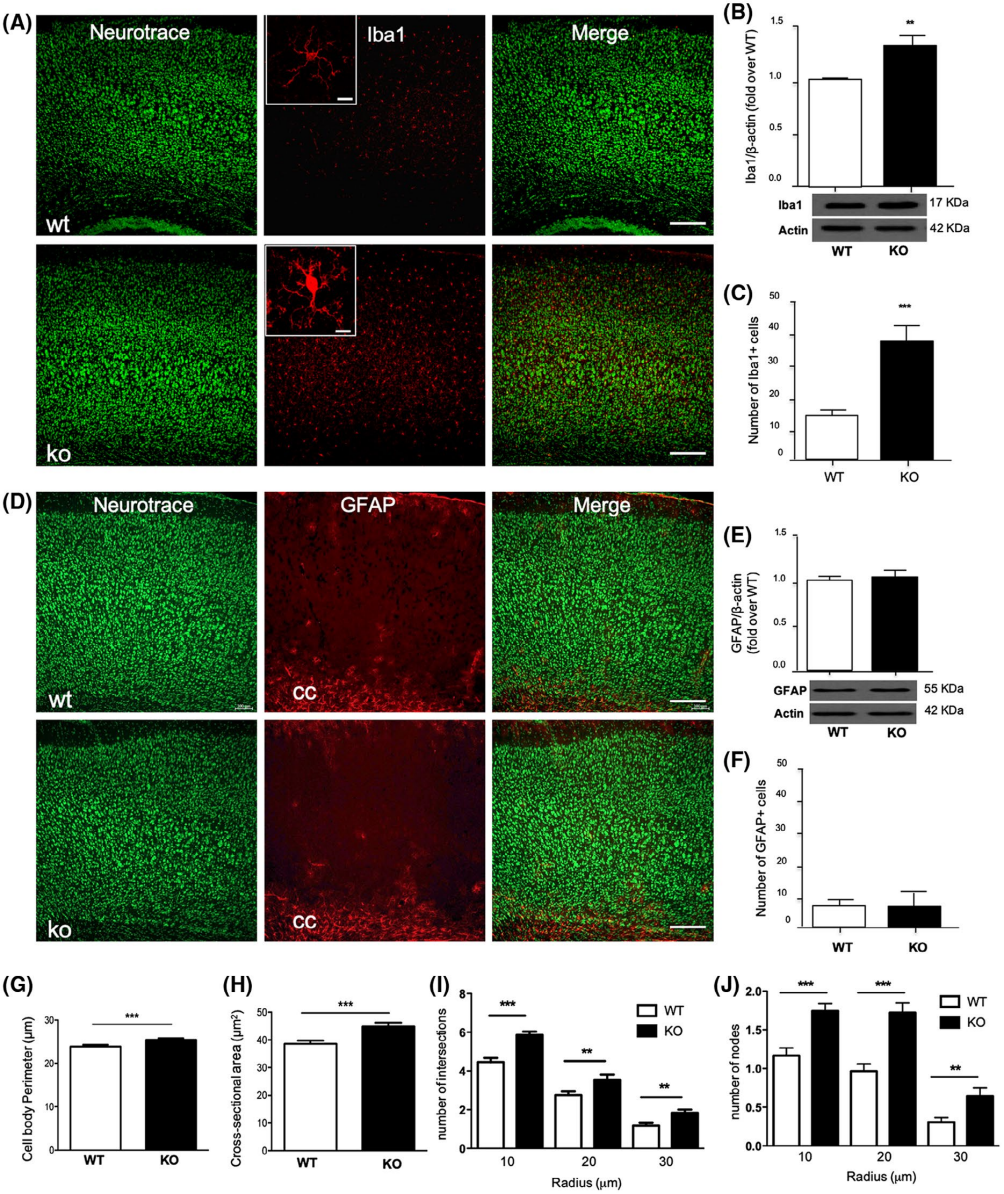
DHA deficiency alters microglia response without affecting astrogliosis. A, Immunofluorescence staining of microglia (Iba1) in the cerebral cortex of WT and KO mice. Double‐labeled and merged confocal images of Iba1 (red) plus NeuroTrace Nissl staining (green) (scale bars = 100 μm). Insets: Single‐labeled images of Iba1 staining (scale bars = 10 μm). B, Western blotting and its densitometry of Iba1. Immunoblot images are cropped from different parts of the same gel. C, Histogram of the number of Iba1 positive cells in the cerebral cortex of WT and KO mice. D, Immunofluorescence staining of astrocytes (GFAP) in the cerebral cortex of WT and KO mice. In the lower portion of the sections it is well visible also the corpus callosum (cc). Double‐labeled and merged confocal images of GFAP (red) plus NeuroTrace Nissl staining (green) (scale bars = 100 μm). E, Western blotting and its densitometry of GFAP. Immunoblot images are cropped from different parts of the same gel. F, Histogram of the number of GFAP positive cells in the cerebral cortex of WT and KO mice. G‐K, Histograms of the Sholl analysis of microglia in the cerebral cortex of WT and KO mice showing the perimeter (G) and the cross‐sectional area of the Iba1 + cells (H), the number of intersections (I), and the number of nodes/branch points at the different radii (J). All results are mean ± SEM or representative of six animals per group (WT and KO) *P < .05, **P < .01, and ***P < .001 vs WT by unpaired Student's t test

**Figure 5:**
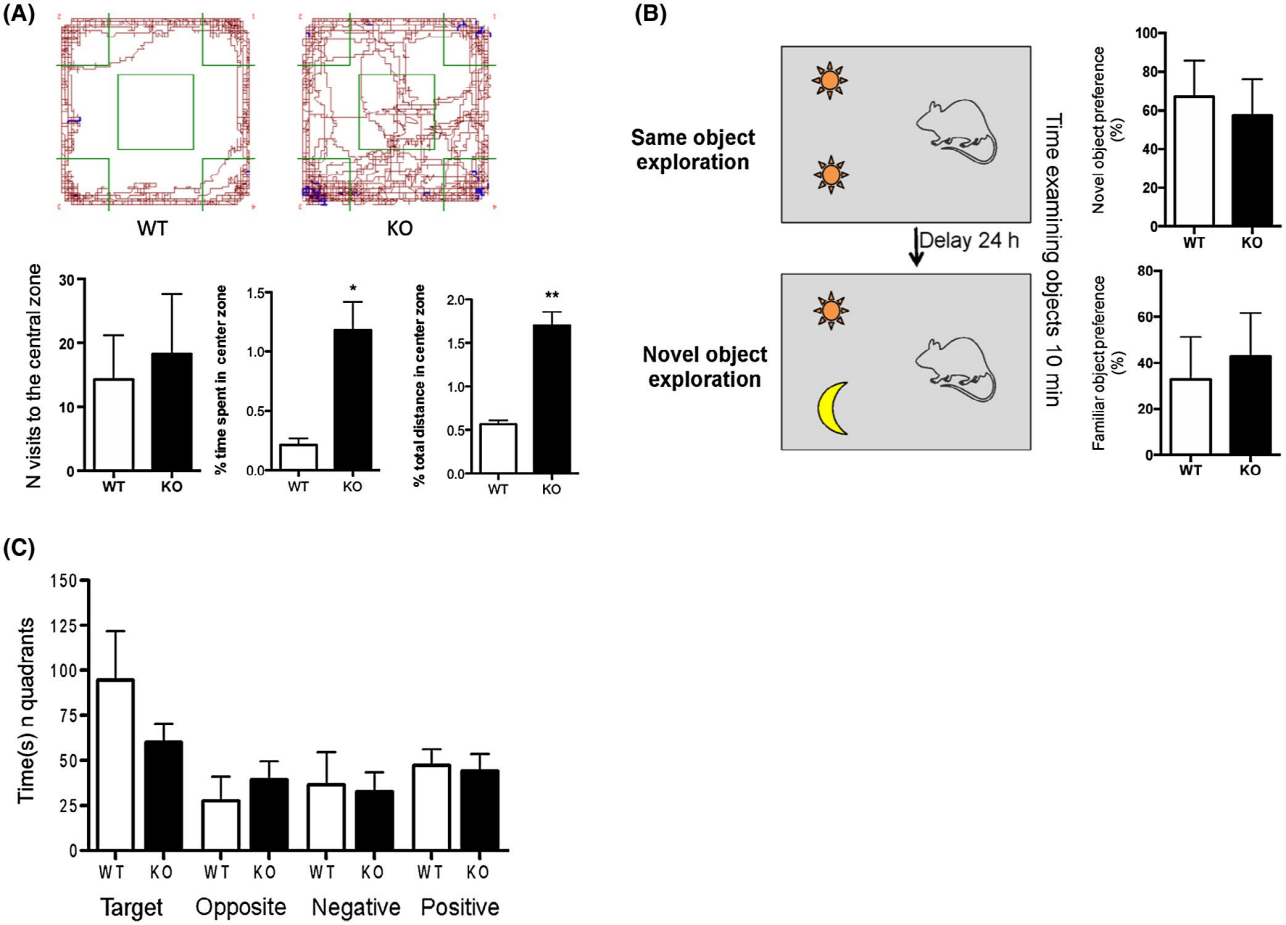
DHA deficiency does not alter behavioral functions. A, Individual exploration trajectories showing the change in the exploratory activity of WT and KO mice. Brown lines indicate locomotion (horizontal movements), blue lines indicate rears (vertical movements), and green lines indicate center area and corners. Exploratory activity was calculated as the number of visits to the center zone, percentage of time spent in the center zone, and percentage of distance in the center zone. Data are shown as mean ± SEM of 12 WT and 16 KO mice and analyzed by the unpaired Student's t test. B, Working memory consolidation with two objects (novel and familiar) evaluated as the percentage of time spent at exploring the objects. Data are shown as mean ± SEM of seven animals per group (WT and KO) and analyzed by the unpaired Student's t test. C, Spatial and learning memory evaluated as the ability of animals to seek out for escape holes (time spent in quadrants of target, opposite, negative or positive holes). Data are shown as mean ± SEM of 8 WT and 7 KO and analyzed by the unpaired Student's t test

Since the presence in the brain of inflammatory markers like cytokines and iNOS is linked to glial cells’ activation, we next investigated the effect of endogenous DHA deficiency on astrocytes and microglia functional state and their morphology in the cerebral cortex. Although quantitative analysis of cortical GFAP protein level and GFAP positive and reactive astrocytes did not show any significant differences between KO and WT mice (Figure 4D‐F), we observed a significant increase of Iba1 protein level and in the number of Iba1 + cells in the cerebral cortex of KO mice compared to WT (Figure 4A‐C), suggesting that DHA‐deficient mice displayed not only an increased number of microglia, but these cells displayed a different morphological state. Interestingly, when closely observing the morphology of Iba1 + microglia, we noticed that the microglia of KO mice showed different morphological features. Thus, we performed Sholl analysis and found that the cerebral cortex of KO mice microglia displayed a higher degree of complexity and ramification compared to those of WT, presenting a larger cell body (increase perimeter and cross‐sectional area) (Figure 4A, inset) as well as an increased number of intersections and nodes of their processes (Figure 4G‐J), suggesting the different state of microglia in KO mice compared to WT. To show that the reduced expression of genes associated with neural plasticity and the exacerbated inflammation observed in KO mice might have a physiological impact in this mouse model deficient for DHA synthesis, we also performed some behavioral analyses. Thus, to examine responses to an unfamiliar environment, Elovl2 WT and KO mice were evaluated in the open field test. Our results show that even if no statistical difference was present in the number of visits to the center zone, the percentage of time spent and the distance covered in the center zone were statistically significant between the two groups of animals (Figure 5A). The same set of animals was also subjected to the NOR test, in which KO animals show a tendency to spend less time, although not significant, at the novel object compared to WT (Figure 5B), suggesting no deficit in object memory. Next, we also evaluated the spatial learning and memory with the Barnes Maze test in a small group of female mice and, also in this case, no differences between WT and KO were observed in the learning curve and in the amount of time spent in the target quadrant (Figure 5C). These results suggest that even if the deficiency of endogenous DHA affects inflammatory and neuroplasticity markers, it does not overall influence behavioral functions.

### 3.4 Neuroplasticity and neuroinflammatory markers are restored by dietary DHA supplementation

**Figure 6:**
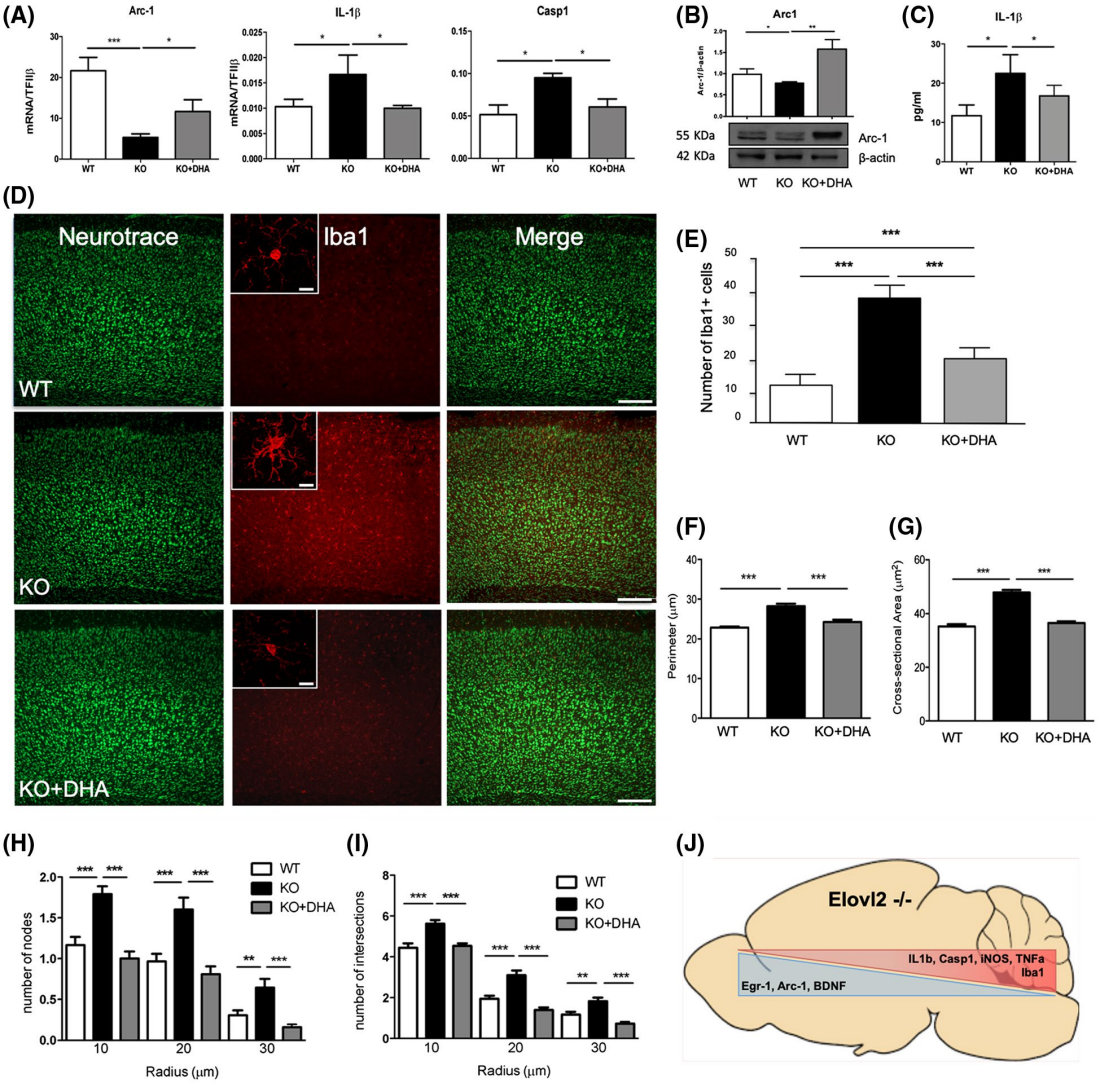
Dietary supplementation with DHA restores neuroplasticity and microglia status in the cerebral cortex. A, mRNA expression of Arc‐1, IL‐1β, and Casp1 in the cortex by qRT‐PCR. Data are shown as mean ± SEM of four animals per group (WT, KO, and KO + DHA), each in duplicate. ***P < .001 vs WT and *P < .05 vs KO by one‐way ANOVA. B, Protein expression of Arc‐1 by western blotting and (C) level of IL‐1β by ELISA. Data are shown as mean ± SEM of four animals per group (WT, KO, and KO + DHA), each in duplicate. ***P < .001 vs WT and *P < .05 vs KO by one‐way ANOVA followed by Bonferroni *post‐hoc* test. D, Immunofluorescence staining of microglia in the cortex of WT, KO, and DHA‐supplemented KO (KO + DHA) mice. Double‐labeled and merged confocal images of Iba1 (red) plus NeuroTrace Nissl staining (green) in the cerebral cortex (scale bars = 100 μm). Inset: Single‐labeled images of Iba1 staining (scale bars = 10 μm). E, Histogram of the number of Iba1 positive cells in the cerebral cortex. Data are shown as mean ± SEM of six animals per group (WT, KO, and KO + DHA). ***P < .001 and **P < .01 by one‐way ANOVA followed by the Bonferroni *post‐hoc* test (F‐I). Histograms of the Sholl analysis of microglia in the cerebral cortex of WT, KO, and KO + DHA mice showing the perimeter (F) and the cross‐sectional area of the cells (G), the number of intersections (H), and the number of nodes/branch points of intersections at the different radii (I). Results are mean ± SEM or representative of six animals per group (WT, KO, and KO + DHA). **P < .01 and ***P < .001 by one‐way ANOVA followed by Bonferroni *post‐hoc* test. J, Schematic representation of DHA deficiency‐induced impact on brain plasticity and inflammation markers

Given the alteration observed in the cerebral cortex of KO mice in terms of expression of neuroplasticity and neuroinflammation markers, we next questioned whether a short‐term dietary supplementation with DHA could restore some of these markers in the cerebral cortex of KO mice. In particular, the reintroduction of DHA in KO mice significantly increased the mRNA expression levels of Arc‐1 and reduced that of IL‐1β and Casp1 compared to KO, with IL‐1β and Casp1 also showing comparable levels to those of WT mice (Figure 6A). This DHA‐induced effect was also corroborated analyzing the protein levels of Arc‐1 and IL‐1β (Figure 6B‐C), with the former showing even higher levels of expression compared to WT (Figure 6B), while the levels of the latter were equal to WT mice (Figure 6C). Similarly, we observed a marked and significant decrease in the number of Iba1 + cells in DHA‐supplemented KO mice compared to KO mice (Figure 6D‐E), showing that the number of Iba1 + cells was comparable to WT mice (Figure 6F‐I). Of note, the DHA‐deficiency‐induced changes in the morphology of microglia observed in KO mice were also counteracted upon DHA supplementation, with microglia presenting a morphological state similar to that observed in WT mice (Figure 6D,F‐I). These findings were supported by the evidence that DHA supplementation significantly restored the brain levels of DHA (Figure S1A), suggesting a key role of DHA in mediating our observed effects. Of note, DHA supplementation did not impact on body weight (Figure S1B) or food intake (Figure S1C).

Furthermore, to rule out the possibility of stain infiltrated Iba1 + monocytes/macrophages from the periphery, we also performed double immunofluorescence combining Iba1 with TMEM‐119, a more specific marker of resident microglia.^41^ Confocal analysis of Iba1/TMEM‐119 confirmed that the number of microglia TMEM‐119 + was significantly higher in KO mice and reduced upon DHA reintroduction, but also demonstrated that both markers perfectly colocalize (Figure S2), thus confirming that the DHA‐induced modulation of neuroinflammation more likely involves only resident microglia.

## 4 DISCUSSION

The beneficial properties of dietary ω‐3 polyunsaturated fatty acids (PUFA), particularly of DHA, have been recognized for decades and their metabolic dysfunction has been linked to a range of diseases including inflammatory and neurodegenerative disorders.^42^, ^43^ Indeed, DHA is an important component of neural membranes, regulating membrane fluidity, permeability, and viscosity in synaptic membranes as well as playing a key role in modulating neurotransmission and synaptic function and plasticity.^44^ However, although its crucial function in brain processes is undisputed, the specific role of the endogenous synthesis of DHA in the brain and how this affects neural plasticity and inflammation is still obscure.

Thus, in this study, we thoroughly investigated for the first time whether the endogenous synthesis of DHA can impact on markers of brain plasticity and inflammation. For this purpose, we used mice deficient for Elovl2 (Elovl2^−/−^), a key enzyme involved in the synthesis of DHA.^45^ Our group has previously shown that these mice are characterized by severely reduced DHA levels in testis, serum, liver, mammary gland and white adipose tissue^6^, ^26^, ^44^ as well as by a pro‐inflammatory status of innate immune cells.^25^ In the present work, we collected the unprecedented evidence of acyl‐CoA levels in brain by targeted lipidomics and we demonstrated that Elovl2‐mediated endogenous DHA synthesis plays a role in the expression of several brain plasticity and inflammatory markers. Although, to date, it is unclear why brain phospholipids are specifically enriched in DHA and low in DPA and EPA, the fact that DHA enrichment is conserved across species^46^, ^47^ suggests that there is a highly specific requirement for this essential fatty acid in neuronal membranes. Indeed, we found a significant downregulation of several neural plasticity factors (Arc‐1, Egr‐1, and BDNF) and a concomitant upregulation of key inflammatory markers (TNF, IL‐1β, iNOS, and Casp1) in Elovl2^−/−^ mice. Interestingly, variations of such markers were particularly evident in the cerebral cortex and not in the total brain, confirming the essential role of this fatty acid in the brain region where it is mostly abundant and corroborating our previous results demonstrating the unique role of Elovl2 in the formation of DHA in the brain.^26^ Our data are in line with previous findings demonstrating that the expression of these markers as well as their related learning and memory functions is modulated by omega‐3 fatty acids in the adult brain.^36^, ^48^, ^49^

However, although decreases in BDNF and Arc‐1 expression are commonly linked to the development of long‐term cognitive and behavioral alterations^11^, ^13^, ^50^, ^51^ and their reduction in the prefrontal cortex are associated with depression,^50^ the mechanism underlying brain DHA reductions and behavioral alterations are still a matter of debate. In addition, considering the substantial evidence regarding the importance of DHA during brain development and on resulting cognitive functions, further investigations are needed to assess a functional relationship between cognitive/behavioral alterations and DHA deficiency in Elovl2^−/−^ mice.

Of note, it is now well established that the brain has fundamental interactions with the immune system.^52^ One of the most exciting aspects of such neuro‐immune communication is that the immune system actively participates in brain functions and synaptic plasticity. In particular glial cells have emerged as active players of synapses, able to shape synapse structure and function.^53^ Indeed, we observed a significant upregulation of several genes involved in neuroinflammation such as iNOS, caspase‐1, and pro‐inflammatory cytokines (IL‐1β and TNF) in the cerebral cortex of DHA‐deficient mice; such high and sustained pro‐inflammatory status is detrimental for both neurons and glial cells, resulting in cell dysfunction or death^54^, ^55^ as well as in the pathogenesis of several cognitive disorders.^56^, ^57^, ^58^ Of note, several cytokines have been reported to suppress synaptic plasticity by suppressing Arc expression in a BDNF‐dependent manner and the latter, together with Egr‐1, is a transcriptional regulator of Arc, thus accounting for our marked changes in Arc‐1 at both mRNA and protein level, while Erg‐1 and BDNF only show changes in their mRNA but not protein. However, the notion that pro‐inflammatory cytokines are only expressed in the brain in response to pathological stimuli has been challenged by emerging data, indicating that certain cytokines can also play an active role in synaptic plasticity in physiological condition.^59^

Yet, the ability of fatty acids to modulate both inflammation and synaptic functions is coming to the foreground and alterations of their availability, either due to insufficient dietary consumption or impaired metabolism, might play a role in the pathogenesis of several cognitive disorders.^38^ In this regard, significant differences in the synaptic proteome of DHA‐adequate and DHA‐deficient mouse brains have been reported^60^ providing a molecular basis for the significant impact of nutritional DHA on some brain functions such as learning and memory. The evidence that free DHA can enter the brain through the mfsd2a and that this transporter is regulated both in Elovl2 mice and upon DHA supplementation suggests not only that Major Facilitator Superfamily Domain containing 2a (Mfsd2a) plays a compensatory role to balance the DHA levels in the brain of Elovl2 mice but also that exists a dynamic interplay between DHA synthesis and DHA uptake in the control of systemic levels.

Intriguingly, glial cells play a crucial role in the regulation of both inflammatory and brain plasticity processes.^61^, ^62^ The fact that our findings revealed the alteration of microglia, but not of astrocytes, in Elovl2^−/−^ mice, suggests that this specific resident immune cell of the brain is specifically affected by DHA deficiency. In particular, not only did we find that the number of Iba1 + cells in the cerebral cortex is increased in DHA deficient mice, but also that its morphology undergoes significant changes. Microglia are dynamic cells whose morphological changes seem to be associated with their functional activities, whereby ramified microglia act as surveying cells, whereas hyper‐ramified microglia are characterized by increased secretion of pro‐inflammatory mediators.^62^ This suggests that microglia might be responsible for the up‐regulation of iNOS, IL‐1β, and TNF observed in Elovl2^−/−^ mice. Although our findings do not clarify the precise cellular source of cytokines, however, our results suggest a temporal correlation between the microglia morphological changes and cytokines production. More studies are required to establish the cellular source of cytokines and, in particular, to demonstrate that their production can be attributed to microglia.

Furthermore, since microglia also play a role in synaptic remodeling and neuronal plasticity,^61^ it is also possible that such activated inflammatory status is somehow associated with the downregulation of markers of synaptic plasticity in Elovl2^−/−^ mice. This is particularly important since the brain and the immune systems are engaged in a bidirectional crosstalk and our data suggest that microglia might engage in mediating such a complex interaction. Furthermore, the fact that such changes are even influenced by DHA deficiency alone, in absence of any brain insult, suggests that ω‐3 essential fatty acid is a master regulator of brain responses and an intriguing interface between “mind” and “body.” However, only a few groups have evaluated the consequences of PUFAs manipulation on microglia in vivo and in cerebral cortex of adult mice after a pathological event.^63^, ^64^ Accordingly, the evidence that reintroducing DHA in the diet of Elovl2^−/−^ mice restored the expression levels of key neuroinflammatory (IL‐1β and Casp‐1) and neuroplasticity (Arc‐1) markers as well as prevented the microglia DHA deficiency‐induced morphologic changes in the cerebral cortex further corroborates our findings. Of note, whether such DHA‐induced neuroprotective effects are mediated by this fatty acid alone or by its derived and specialized pro‐resolving mediators (SPMs) have yet to be unraveled. The discoveries that several DHA‐derived SPMs, such as resolvins or neuroprotectins, play critical roles in modulating microglia responses as well ad in the modulation of reactive species^24^, ^65^, ^66^, ^67^ is suggestive of their possible involvement in controlling neuroplasticity and in representing a link between the immune and the central nervous systems. In addition, our findings show that even in the absence of inflammatory stimuli or without the induction of specific diseases, systemic DHA deficiency induces a basal inflammatory activity, which is characterized by an overall upregulation of several inflammatory markers; however, this only has a little impact on behavioral functions. Further experiments to prove whether such DHA‐deficient mice are more responsive to inflammatory events or are more susceptible to chronic neuroinflammatory diseases or cognitive disorders are needed. Yet the evidence that a ω‐3 deficient diet is associated with exacerbated inflammation and memory deficits in another mice model exposed prenatally to LPS^68^ suggests that DHA‐induced profound and macroscopic cognitive alterations appear mostly under inflammatory conditions and account for the discrete behavioral dysfunctions observed here. It is important to add that since the Iba1 microglia marker can also stain macrophages migrated from the periphery, TMEM‐119/Iba1 colocalization excludes the possibility that endogenous DHA synthesis may also impact recruitment and activation of infiltrated cortical monocytes/macrophages.

Furthermore, we cannot exclude also the possibility that our DHA‐induced effects on microglia are mediated by a direct action on these cells or are indirectly mediated by influencing other molecules released by astrocytes or even neurons, calling for future mechanistic studies unraveling the cellular and molecular signaling pathways. The unraveling of these issues would also be crucial to understand whether the changes in markers of brain plasticity and neuroinflammation take place within the same cells and are linked to one another and what are the mechanisms underlying their interaction.

In conclusion, as summarized in Figure 6J, this study has highlighted the important role of the DHA‐generating Elovl2 enzyme in modulating brain neuroplasticity and inflammation, with conceivable and potential implications in affecting CNS homeostasis. Indeed, our findings suggest that the enzymatic machinery that leads to DHA synthesis is involved, at least in part, in the maintenance of brain functions and supports the notion that diets rich in DHA could be sufficient to activate some compensatory pathways that might reduce neuroinflammation.

## CONFLICTS OF INTEREST

The authors declare no conflict of interest.

## AUTHOR CONTRIBUTION

E. Talamonti, M.T. Viscomi, and V. Chiurchiù conceived and designed the study; E. Talamonti, V. Sasso, H. To, R.P. Haslam, K. Pernold, and A. Asadi performed and analyzed the data; J.A. Napier, B. Ulfhake, T. Hessa, A. Jacobsson, V. Chiurchiù, and M.T. Viscomi provided reagents and tools; V. Chiurchiù, M.T. Viscomi, and E. Talamonti wrote the paper.
