# Supplementary material for: Impairment of DHA synthesis alters the expression of neuronal plasticity markers and the brain inflammatory status in mice
Source: FASEB J. 2019 Dec 11;34(2):2024–40. doi: 10.1096/fj.201901890RR (PMC7384056; doi:10.1096/fj.201901890RR)
Supplement: Supplementary file 1 [file FSB2-34-2024-s001.docx]

***Impairment of DHA synthesis alters the expression of neuronal plasticity markers and the brain inflammatory status in mice***

Talamonti E^1,2#^, Sasso V^3^, To H^1^, Haslam RP^4^, Napier JA^4^, Ulfhake B^5^, Pernold K^5^, Asadi A^2^, Hessa T^1^, Jacobsson A^2^, Chiurchiù V^6,7*^, Viscomi MT^8*#^

*^1^Department of Biochemistry and Biophysics, Stockholm University, Sweden*

*^2^Department of Molecular Biosciences, The Wenner-Gren Institute, Stockholm University, SE-10691 Sweden*

*^3^Laboratory of Experimental Neurorehabilitation, IRCCS Santa Lucia Foundation, Rome, Italy*

*^4^Department of Plant Science, Rothamsted Research, Harpenden, United Kingdom*

*^5^Department of Neuroscience, Karolinska Institute SE-171 77 Stockholm, Sweden*

*^6^Department of Medicine, Campus* Bio-Medico *University of Rome, Rome, Italy*

*^7^Laboratory of Neurochemistry of Lipids, IRCCS Santa Lucia Foundation, Rome, Italy*

*^8^* *Istituto di Istologia ed Embriologia, Università Cattolica del S. Cuore, Rome, Italy*

**Supplementary Fig.1. DHA levels in the brain, body weight and food intake after dietary DHA supplementation.** Levels of DHA (22:6 n-3) in the brain (**A**), body weight (**B**) and food intake (**C**) of WT and KO mice fed standard chow diet (no DHA) and KO mice fed with a DHA-enriched diet. Results are mean ± SEM or representative of 4-5 animals per group (WT, KO and KO+DHA). **p<0.01, ***p< 0.001 by one-way ANOVA followed by Bonferroni post-hoc test.


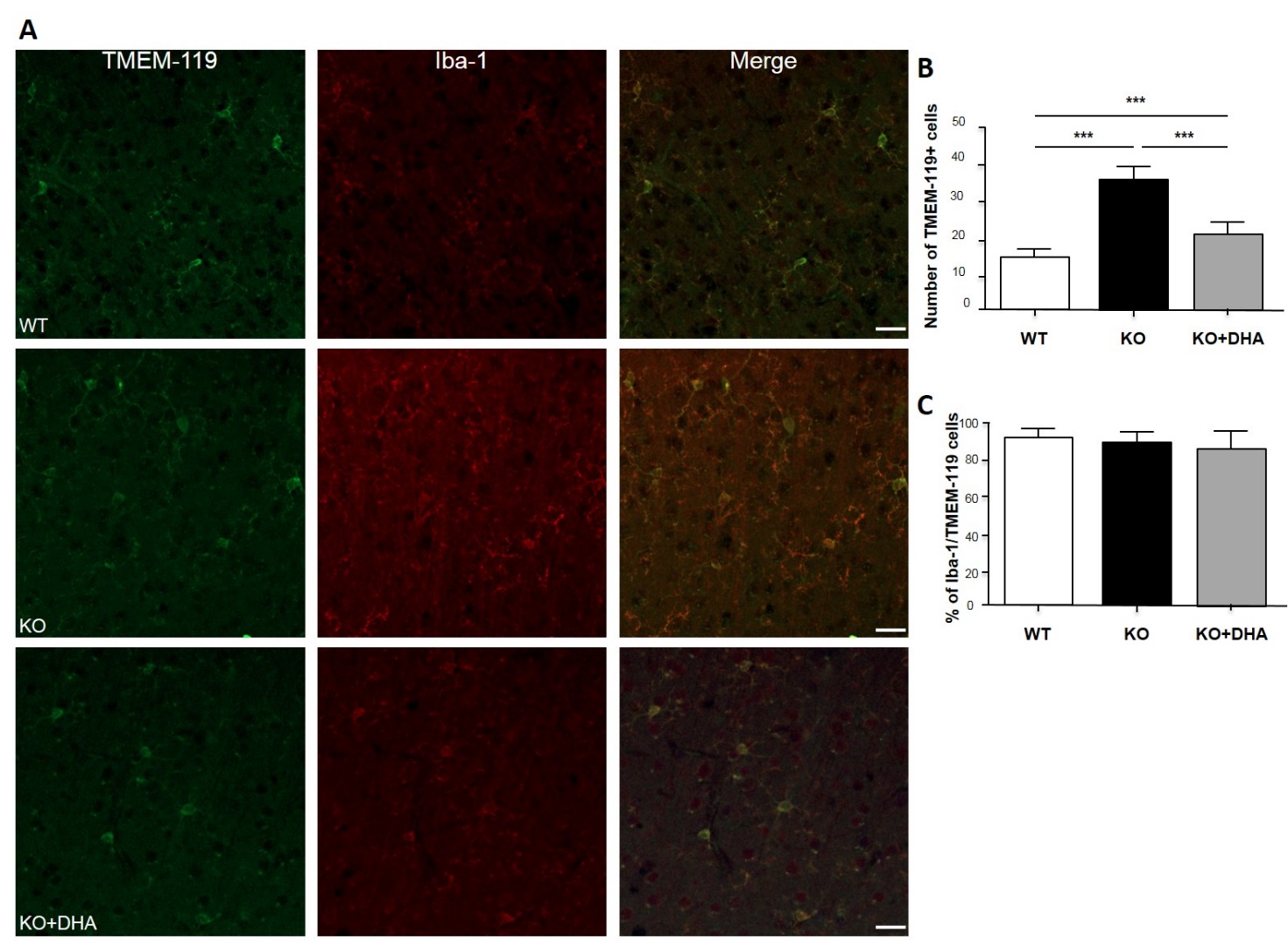


**Supplementary Fig.2. DHA deficiency does not affect the recruitment and activation of infiltrated monocytes/macrophages.** (**A**) Double immunofluorescence staining of TMEM-119 (green), a specific marker of resident microglial cells, and Iba-1 (red), a marker of both infiltrated monocytes/macrophages from the periphery as well as resident microglial cells, in cortex of WT, KO and KO+DHA mice. Scale bars =20 μm. (**B**) Histogram of the number of TMEM-119 positive cells in the cerebral cortex of WT, KO and KO+DHA mice. (**C**) Histograms of TMEM-119/Iba-1 co-localization in cortex of WT, KO and KO+DHA mice expressed as percentage. Results are mean ± SEM or representative of 6 animals per group (WT, KO and KO+DHA). ***p< 0.001 by one-way ANOVA followed by Bonferroni post-hoc test.
